# Lightweight Fine-Tuning for Pig Cough Detection

**DOI:** 10.3390/ani16020253

**Published:** 2026-01-14

**Authors:** Xu Zhang, Baoming Li, Xiaoliu Xue

**Affiliations:** 1 Department of Agricultural Structure and Bioenvironmental Engineering, College of Water Resources and Civil Engineering, China Agricultural University, Beijing 100083, China; 20191026@bjut.edu.cn (X.Z.);; 2 Beijing Engineering Research Center of Precision Measurement Technology and Instruments, Beijing University of Technology, Beijing 100124, China; 3 Key Laboratory of Agricultural Engineering in Structure and Environment, Ministry of Agriculture and Rural Affairs, Beijing 100083, China; 4 Beijing Engineering Research Center for Animal Health Environment, Beijing 100083, China

**Keywords:** pig cough recognition, PANNs-CNN14, TFDS, transfer learning, early warning model

## Abstract

Respiratory diseases pose a significant threat to intensive pig farming, and cough recognition serves as a key indicator for early intervention. However, its practical application is constrained by the scarcity of labeled samples and the complex acoustic conditions of farm environments. To address these challenges, this study proposes a lightweight pig cough recognition method based on a pre-trained model. By freezing the backbone of a pre-trained audio neural network and fine-tuning only the classifier, our approach achieves effective knowledge transfer and domain adaptation with very limited data. We further enhance the model’s ability to capture temporal–spectral features of coughs through a time–frequency dual-stream module. On a dataset consisting of 107 cough events and 590 environmental noise clips, the proposed method achieved an accuracy of 94.59% and an F1-score of 92.86%, significantly outperforming several traditional machine learning and deep learning baseline models. Ablation studies validated the effectiveness of each component, with the model attaining a mean accuracy of 96.99% in cross-validation and demonstrating good calibration. The results indicate that our framework can achieve high-accuracy and well-generalized pig cough recognition under small-sample conditions. The main contribution of this work lies in proposing a lightweight fine-tuning paradigm for small-sample audio recognition in agricultural settings, offering a reliable technical solution for early warning of respiratory diseases on farms. It also highlights the potential of transfer learning in resource-limited scenarios.

## Simple Summary

Respiratory diseases in pigs not only threaten animal health but also raise significant welfare concerns in modern farming. Early detection of symptoms such as coughing is essential for timely health management and improving animal welfare. This study tackles the challenge of automatically recognizing pig coughs under small-sample conditions. We propose a transfer learning-based fine-tuning approach using the PANNs-CNN14-TFDS network, which preserves pre-trained acoustic knowledge by freezing the backbone and only lightly tuning the fully connected layers. Experiments confirm that our model achieves high accuracy and strong generalization even with limited data. The approach provides a practical and efficient tool for real-time monitoring and early warning of respiratory diseases, thereby supporting both health management and welfare-oriented pig production.

## 1. Introduction

In large-scale pig farming, respiratory diseases pose a significant threat to herd health, leading to substantial economic losses and hindering the sustainable development of the livestock industry. Therefore, early and accurate detection of such diseases is critical for implementing timely interventions, reducing mortality rates, and minimising antibiotic usage. As a key clinical manifestation of respiratory infections, coughing functions not only as a physiological defence mechanism but also as a vital early-warning indicator [1,2]. Through clinical observations, Ferrari et al. [3] further confirmed that the frequency and duration of coughs are markedly higher in sick pigs than in healthy pigs, and these coughs exhibit distinct acoustic characteristics [4]. This acoustic specificity provides a theoretical foundation for developing artificial intelligence-based automatic cough recognition systems, making such technologies powerful tools for the early detection of respiratory diseases.

However, in real pig housing environments, the noise from ventilation systems, feeding equipment, manure removers, and other pig vocalizations extensively overlaps with cough signals in the time–frequency domain [5,6,7], resulting in an extremely low signal-to-noise ratio. Moreover, due to the high cost of professional annotation, individual farm sites often yield fewer than one hundred labeled samples, leading to a small-sample generalization bottleneck.

To address the challenge of noise robustness, research has evolved from traditional machine learning methods to deep learning approaches. Early studies employed traditional machine learning algorithms such as support vector machines (SVMs) [8,9,10] and hidden Markov models [11,12,13,14] for pig cough recognition. Although effective to a degree, these methods often rely on manual feature engineering (e.g., Mel-frequency cepstral coefficients [MFCCs]) and show limited robustness to noise interference. In recent years, deep learning models have demonstrated superior feature extraction capabilities. The pioneering work of Moshou et al. [14] first applied neural networks to pig cough recognition. Subsequent studies explored various architectures, including deep belief networks [15], backpropagation neural networks [16,17], and convolutional neural networks (CNNs) [18,19], with some introducing attention mechanisms [20] or deep regression networks [21,22] to enhance feature discrimination and speech enhancement. Nonetheless, when applied to the pig farming context, these models—typically trained from scratch with fixed architectures—often fail to overcome the small-sample bottleneck. They tend to overfit the limited training data, capturing noise and spurious patterns rather than the underlying true distribution, which results in poor generalisation to unseen data. This limitation severely constrains the reliability of early-warning systems in practical deployments.

To overcome these challenges, this study proposes a novel and parameter-efficient transfer learning framework designed specifically for the small-sample regime. The core of our approach is a lightweight dual-stream architecture that strategically combines a frozen, pre-trained audio backbone (PANNs-CNN14) with a newly introduced Time–Frequency Dual-Stream (TFDS) module. This design yields three key contributions: (1) It enables effective knowledge transfer and domain adaptation by leveraging general-purpose acoustic representations from AudioSet dataset [13] while requiring updates to less than 0.4% of the backbone’s parameters. (2) The dedicated TFDS module explicitly models the global time-frequency patterns inherent to cough sounds, a feature poorly captured by standard architectures. (3) It establishes a practical and robust learning paradigm that mitigates overfitting and ensures strong generalization on minimal data. Collectively, this work provides a reliable, efficient technical solution for real-time respiratory disease monitoring in resource-constrained farming environments.

The remainder of this paper is organised as follows: Section 2 describes the materials and methods, including data acquisition and feature extraction processes, the architecture of the proposed model, and the training strategy. Section 3 presents the experimental results and analysis, including performance comparisons, generalisation verification, reliability evaluation, and ablation studies. Section 4 discusses the advantages of the proposed method and the impact of hyperparameters, as well as its limitations and potential applications. Finally, Section 5 presents the conclusions of this study.

## 2. Materials and Methods

### 2.1. Data Acquisition System

The experimental data were collected from JiaHeng Pig Farm, located in Chenzhou City, Hunan Province, China. The target area was a pigsty measuring 3.2 × 4.5 m. Data collection was conducted in September 2023, using three Jiuzhou Black pigs aged approximately 105–110 days. Based on visual observations, one pig exhibited continuous coughing and was diagnosed as ill by professional breeders, while the remaining two pigs were healthy and displayed no coughing symptoms.

**Figure 1:**
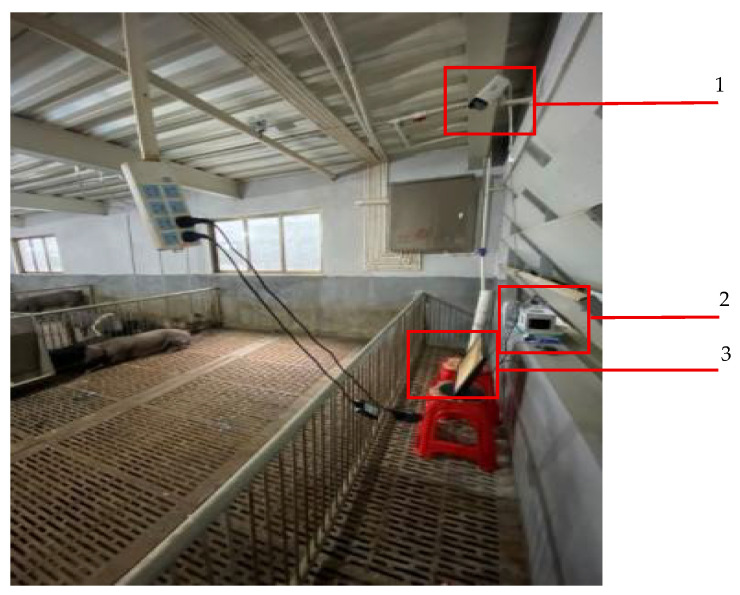
Experimental Setup Layout. (1 Camera, 2 Network Video Recorder, 3 Monitor).

**Figure 2:**
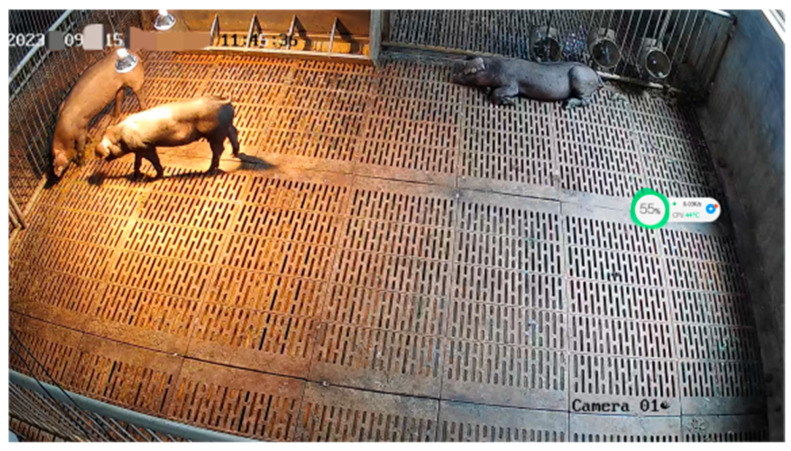
Photographs of Experimental Subjects.

Audio signals were extracted from video recordings captured using a Hikvision DS-IPC-B12HV3-IA bullet camera (Hangzhou Hikvision Digital Technology Co., Ltd., Hangzhou, China), which was mounted on the side above the pigsty at a height of 2.3 m from the ground using a wall-mounted installation method. The layout of the experimental setup and photographs of the test subjects are shown in Figure 1 and Figure 2, respectively.

The primary frequency range of pig coughs lies between 150 Hz and 5 kHz, with the dominant frequency band—where most of the energy and characteristic features are concentrated—typically ranging between 500 Hz and 2 kHz. The 8-kHz audio sampling rate of the camera satisfies the Nyquist criterion, adequately capturing the dominant frequency range of cough sounds. A total of 72 h of continuous audio data were collected. After resampling, a total of 107 cough events (each lasting 0.2–0.5 s) and 590 environmental-noise segments (each lasting 1–3 s) were extracted as valid samples. The dataset was then divided into a training set (487 samples), a validation set (105 samples), and a test set (105 samples).

### 2.2. Feature Extraction Based on the Log-Mel Spectrogram

In continuous audio recordings from pig farms, pig coughs are typically characterised by short-term, high-frequency acoustic energy mutations. To effectively capture these mutations, time-domain audio signals must be mapped to feature spaces capable of highlighting such energy mutations.

Let the original audio signal be represented as $s\left(t\right)$, where t=0,1,…N−1

(1) $$N=f_{0}\times T=8000$$

Here, *N* is the number of sampling points, $f_{0}$ is the sampling rate, and *T* is the uniformly truncated duration.

A short-time Fourier transform is performed to obtain the linear amplitude spectrum, as expressed in Equations (2) and (3):

(2) Sk,t=STFTst∈ℝ^n_fft/2+1×Tframe

(3) $$T_{\mathrm{frame}}=1+(N-n\_\mathrm{fft})/\mathrm{hop}\_\mathrm{length}=1+(8000-256)/128=62$$

where $T_{\mathrm{frame}}$ represents the frame number, n_fft represents the window size, and hop_length represents the frame shift.

Subsequently, a set of Mel filters (*M*) are designed, and the linear magnitude spectrum is projected onto the Mel scale through this filter bank to obtain the energy distribution of each Mel frequency band.

The base-10 logarithm of the energy of each Mel frequency band is computed to yield the Log-Mel spectrogram feature *X* as follows:

(4) X = lnM⋅Sk,t∈ℝ^n_mels×Tframe=ℝ^64×62

Here, n_mels represents the number of Mel filters.

After stacking the audio data within the batch, the output tensor dimension is expressed as:

(5) X∈ℝ^B×n_mels×Tframe=ℝ^8×64×62

where *B* represents the number of audio files within a single batch.

### 2.3. Model Architecture Design Based on the PANNs-CNN14 and TFDS Module

**Figure 3:**
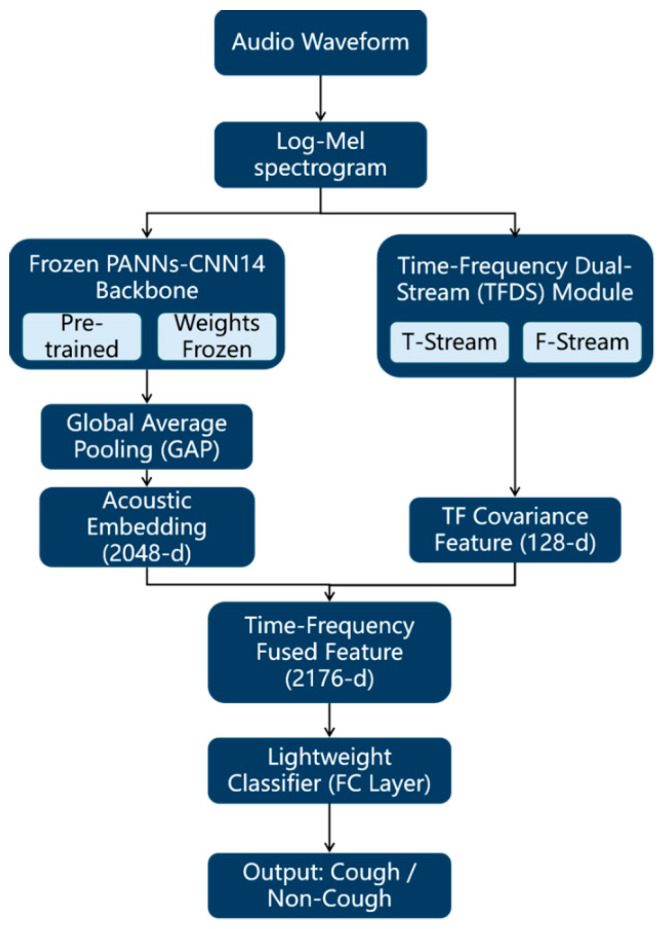
PANNs-CNN14-TFDS Overall Architecture Diagram. **Note:** The gray modules are contained within the blue modules.

To further enhance the generalisation and robustness of the model in complex farm-noise environments, this study proposes an enhanced classification model built upon the PANNs-CNN14 architecture by integrating a parallel TFDS module. The complete model leverages the strong general-purpose acoustic feature extraction capability of the frozen PANNs-CNN14 backbone, while the newly introduced lightweight TFDS branch operates in parallel to specifically capture and fuse complementary temporal and spectral covariance features. This dual-stream fusion improves the modelling of short, impulsive cough-like events. The overall algorithm architecture depicting the interaction between the PANNs-CNN14 backbone and the TFDS module is illustrated in Figure 3.

#### 2.3.1. Pre-Trained PANNs-CNN14 Backbone Design

The PANNs-CNN14 is a deep learning model pre-trained on the large-scale AudioSet dataset released by Google. The model has demonstrated outstanding performance on audio classification tasks, achieving an event localisation ability (mean average precision) of 0.431, a classification discrimination ability (area under the curve (AUC)) of 0.973, and a sensitivity (d-prime) of 2.732.

First, the pre-trained weights of the PANNs-CNN14 backbone network are frozen, and its high-dimensional acoustic embedding (*g*) is extracted.

To ensure accurate input to the frozen backbone, the spectrogram must conform to the expected input layout; therefore, the CNN14 network requires a four-dimensional tensor:

(6) x∈ℝ^{B×C×n_mels×Tframe}=ℝ^8×C×64×62

Therefore, before being fed into the network, a dimension expansion is applied to the *X* value (obtained in Section 2.2) as follows:

(7) x = unsqueezeX,dim=1∈ℝ^8×1×64×62

The hierarchical mapping obtained by feeding *x* into CNN14 can be expressed as:

h^0=x 

(8) h^l=ReLUBNConv^lh^l−1, l=1…14

Here,  h^l represents the feature map output of the layer, capturing increasingly abstract acoustic patterns at each level; Conv^l denotes two-dimensional convolution, responsible for extracting local time–frequency features; *BN* represents batch normalisation, which stabilises feature distribution and accelerates convergence; ReLU represents the activation function, introducing the key nonlinear transformation that enhances the expressive power of the model.

Through 14 layers of cascaded feature transformation, the network ultimately compresses spatiotemporal dimensions via global average pooling to distil high-level acoustic representations as follows:

(9) g14= GAPh^14∈ℝ^2048

This operation distils the multi-dimensional feature  h^l into a compact embedding vector $g_{14}$, which contains discriminative acoustic fingerprint information.

#### 2.3.2. Recognition Algorithm Enhanced by the TFDS Module

**Figure 4:**
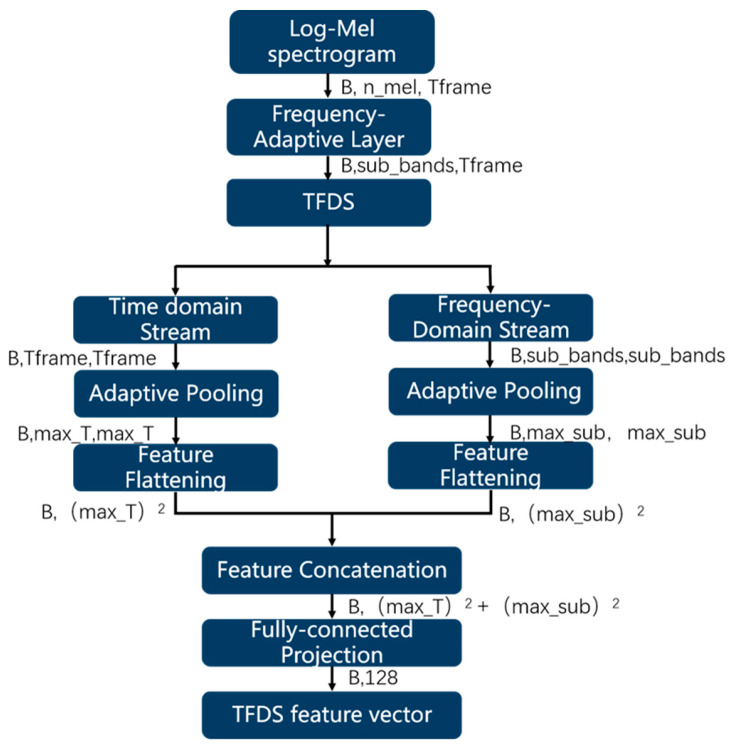
TFDS Model Architecture Diagram.

To further strengthen the generalisation and robustness of the model in complex farm-noise environments, we integrated a parallel TFDS module into the PANNs-CNN14 backbone. This lightweight auxiliary branch fuses temporal and spectral covariance features to improve the modelling of short cough-like pulses. The structure of the TFDS module is shown in Figure 4.

First, frequency-domain adaptation is performed via a 1 × 1 convolution as follows:

(10) Z=ReLUBatchNormConv1DXT∈ℝ^{B×Subbands×Tframe}=ℝ^8×32×62

where *Subbands* denotes the number of sub-bands, which corresponds to the optimised feature dimensionality learnt by the model.

Second, the temporal covariance matrix is computed as follows:

(11) Ct=Z⋅ZT∈ℝ^B×Tframe×Tframe=ℝ^8×62×62

This matrix captures the correlations between different time points within the signal, reflecting the temporal structure characteristics of cough impulses.

Third, the spectral covariance matrix is computed as follows:

(12) Cf=ZT⋅Z∈ℝ^B×Subbands×Subbands=ℝ^8×32×32

This matrix characterises the correlations between different frequency bands, capturing the spectral distribution patterns of cough signals.

To prevent parameter explosion and standardize feature dimensions, adaptive pooling is applied to covariance matrices as follows:

(13) $${C_{t}}^{\prime }=\mathrm{AdaptiveAvgPool}2D\left(C_{t},\left(T_{max},T_{max}\right)\right)$$

(14) $${C_{f}}^{\prime }=\mathrm{AdaptiveAvgPool}2D\left(C_{f},\left(S_{max},S_{max}\right)\right)$$

where $T_{max}=25,S_{max}=16$ are predefined maximum sizes.

The pooled covariance matrices are then flattened and concatenated:

(15) flat=flattenCt′;flattenCf′∈ℝ^B×(T2max+S2max)=ℝ^8×881

Finally, the features are compressed to a hidden dimensionality via a fully connected (FC) layer as follows:

(16)  gtfds=ReLU(Wtfds⋅flat+btfds)∈ℝ^B×H=ℝ^8×128

where H=128 denotes the output dimensionality of the TFDS module, and $W_{tfds}$ and $b_{tfds}$ are the learnable weight matrix and bias vector, respectively.

#### 2.3.3. Lightweight Classifier Design

Based on the fused feature vector *g*:

(17)  gt=g14+gtfds∈ℝ^8×2176

We constructed a lightweight classification network, following a structured three-level paradigm: ‘regularisation, projection, and decision-making’.

(1) Random Regularisation: Dropout Layer

To mitigate the risk of overfitting with small sample sizes, a dropout mechanism (with probability *p* = 0.2) is introduced. This layer generates a binary mask *m* via the Bernoulli distribution, randomly discarding 20% of neuronal connections. The output vector *h* is computed by performing an element-wise multiplication of the input with *m*, subsequently scaling the result by $\frac{1}{1-p}$:

(18) $$h\;=\;\frac{1}{1-p}(m⊙g_{t})$$

where ⊙ denotes element-wise multiplication.

(2) Feature Projection: FC Layer

The regularised vector *h* is then mapped to the logits space through an FC layer. The core function of this layer is to project high-dimensional fused features into a lower-dimensional logits space, providing discriminative signals for the final classification decision.

(19) z=W·h+b, z∈ℝ^2

where the weight matrix W∈ℝ^2176×2 is used to map the output of the Dropout layer to the classification space, the bias vector  b∈ℝ^2 is used to provide the category bias term, and *z* is the un-normalised logit, containing the original discriminative signal of cough/non-cough.

(3) Probability Normalisation: Softmax Decision Layer

To facilitate classification decision-making, the output of the linear transformation is converted into a probability distribution. Next, the probability of the i-th sample being predicted as a cough is determined as follows:

(20) $$p_{i1}=\mathrm{softmax}\left(z_{i1}\right)=\frac{e^{z_{i1}}}{e^{z_{i1}}+e^{z_{i2}}}$$

The probability $p_{i2}$ of not coughing is predicted as

(21) $$p_{i2}=\mathrm{softmax}\left(z_{i2}\right)=\frac{e^{z_{i2}}}{e^{z_{i1}}+e^{z_{i2}}}=1-p_{i1}$$

The final forward propagation formula is expressed as

(22) p= softmax11−pW(m⊙g)+b ∈ℝ^2

### 2.4. Training Strategy

To equip the PANNs-CNN14-TFDS network with the ability to distinguish coughs produced by sick pigs from other environmental sounds, a comprehensive training protocol is required. The following three sub-sections describe, in sequence: (1) the loss function that defines the optimisation target; (2) the back-propagation scheme that computes gradients while maintaining the backbone frozen; and (3) the optimiser hyperparameters and regularisation strategies that convert raw gradients into stable, effective weight updates.

#### 2.4.1. Calculation of the Loss Function

The predicted probabilities satisfy the constraint $p_{i1}+p_{i2}=1$. The true label is expressed in one-hot encoding form as $\left(y_{i1},y_{i2}\right)$, where $y_{i1},y_{i2}\in \left\{0,1\right\}$ and satisfy $y_{i1}+y_{i2}=1$. Specifically, $y_{i1}=1$ denotes a cough sample, while $y_{i2}=1$ corresponds to a non-cough sample.

The forward propagation of the PANNs-CNN14-TFDS network yields a predictive distribution for the cough recognition task. After completing the forward propagation design of the network, the model possesses the full mapping ability from the original audio input to the predicted probability output. However, to enable the model to truly learn the ability to discriminate between the coughs of sick pigs and other sounds, a loss function must be introduced as the guidance of the training process. The loss function quantifies the difference between the predicted probability $p_{i1}$ and the actual label $y_{i1}$, essentially constructing an optimisable mathematical distance metric.

This study adopted the average cross-entropy loss function as the optimisation objective for model training, expressed as follows:

(23) $$L=-\frac{1}{K}\sum_{i=1}^{K}\left[y_{i1}\ln p_{i1}+y_{i2}\ln p_{i2}\right]$$

Here, *K* represents the number of samples. The cross-entropy loss function reflects the distance between the predicted probability distribution of the model and the actual label distribution. A smaller value indicates a better model performance.

#### 2.4.2. Backpropagation

Backpropagation, serving as the core process in neural network training, provides the foundation for the optimisation procedure by computing gradients of model parameters with respect to the loss function. This study adopted a transfer learning fine-tuning paradigm: the pre-trained PANNs-CNN14 backbone network is maintained frozen, while only the parameters of the TFDS module and the final classification layer are optimised. For the Dropout layer, as a stateless mechanism, it directly reuses the binary mask m generated during forward propagation in the backward pass, without maintaining or updating any internal parameters.

**Figure 5:**
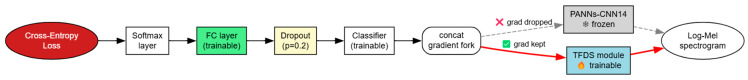
Gradient Calculation Path.

As illustrated in Figure 5, the specific gradient computation path is as follows: gradients originate from the cross-entropy loss function, propagate backward through the Softmax layer, and subsequently compute the parameter gradients of the classification layer. When gradients reach the feature concatenation point—that is, where the outputs of the frozen backbone network and the TFDS module are fused—they bifurcate into two independent gradient streams: one stream propagates backward through the TFDS module, computing and updating all its trainable parameters ($W_{tfds}$, $b_{tfds}$); and the other stream enters the frozen PANNs-CNN14 backbone network. Although gradients flow through this path due to the computational graph, they are actively discarded and not used to update any parameters of the backbone network. This ensures that the general acoustic feature extraction capability acquired through pre-training is fully preserved.

This strategy establishes an efficient dual-stream optimisation architecture that leverages both the general knowledge of the frozen backbone network and the specialised learning of pig cough features through the lightweight TFDS module. Computationally, this approach reduces the number of trainable parameters from approximately 23 million in the backbone network to about 89,000, significantly lowering computational overhead and the risk of overfitting, making it particularly suitable for few-shot learning scenarios.

The gradient of the loss function *L* with respect to the predicted probability *p_i_* can be expressed as

(24) $$\frac{\partial L}{\partial p_{i}}=-\frac{1}{K}\cdot \frac{y_{i}}{p_{i}}\;$$

The gradient of the Softmax layer is expressed as

(25) $$\frac{\partial L}{\partial z_{i}}=\frac{1}{K}(p_{i}-y_{i})$$

The gradient of the FC layer is given by

(26) $$\frac{\partial L}{\partial W}=\frac{\partial L}{\partial z_{i}}\times \frac{\partial z_{i}}{\partial W}=h^{\;T}\frac{\partial L}{\partial z_{i}}=h^{\;T}\frac{1}{K}(p_{i}-y_{i})$$

(27) $$\frac{\partial L}{\partial b}=\frac{\partial L}{\partial z_{i}}\times \frac{\partial z_{i}}{\partial b}=\frac{\partial L}{\partial z_{i}}=\frac{1}{K}(p_{i}-y_{i})$$

#### 2.4.3. Optimisation with Regularisation for Stable Weight Updates

After defining the gradient calculation principle for backpropagation, we designed the model training strategy. A hierarchical progressive strategy is adopted: first, dual regularisation is implemented by combining weight decay and gradient clipping to jointly suppress the risk of overfitting in small-sample scenarios. Subsequently, the Adam optimiser is used to adjust the gradient step size. Ultimately, a complete training system featuring closed-loop optimisation is established.

In the optimiser, the overall gradient $g_{t}$ of the FC layer is the vectorised concatenation of the weight gradient and the bias gradient and is expressed as

(28) gt=vec(∂L∂W)∂L∂b∈ℝ^2×2176+2=ℝ^4354

Here, $vec(\frac{\partial L}{\partial W})$ represents the flattened weight gradient vector.

Weight decay (with a decay coefficient λ = 10^−5^) is added to $g_{t}$ to obtain the new regularised gradient $g_{reg}$:

(29) greg=gt+λvec(W)b∈ ℝ^4354

Here, vec(W) is the flattened weight vector.

Subsequently, gradient clipping is implemented in the gradient space dimension (with a threshold τ = 0.3), mathematically constraining the gradient norm to a safe range to prevent gradient explosion during mini-batch training.

The regularised gradient $g_{reg}$, when expanded, is given by

(30) greg=[greg1,greg2,…,gregn]T∈ℝ^4354

Its gradient norm is expressed as:

(31) $$∥g_{reg}{∥}_{2}=\sqrt{\sum_{k=1}^{n}g_{k}^{2}}$$

where ${\left\|\cdot \right\|}_{2}$ denotes L2 norm operator, used to compute the magnitude of the gradient vector.

This formula represents the calculation of the L2 norm for the vector $g_{reg}$. It quantifies the total length of this regularized gradient vector by taking the square root of the sum of the squares of its components.

Given the threshold, the clipped gradient is determined as:

(32) $$g_{clip}=\left\{\begin{array}{cc} g_{reg} & \mathrm{if}{\left\|g_{reg}\right\|}_{2}\leq \tau \\ \frac{\tau \cdot g_{reg}}{∥g_{reg}{∥}_{2}} & \mathrm{if}{\left\|g_{reg}\right\|}_{2}>\tau \end{array}\right.$$

The Adam optimiser is then used to update the parameters of the FC layer, *W* and *b*.

The first and second moment estimates are calculated as follows:

(33) $$m_{t}=\beta_{1}m_{t-1}+(1-\beta_{1})g_{clip}$$

(34) $$v_{t}=\beta_{2}v_{t-1}+(1-\beta_{2}){g^{2}}_{clip}$$

Here, $m_{t}$ and $v_{t}$ are the first and second moment estimates, respectively; $\beta_{1}$ and $\beta_{2}$ are the hyperparameters of the Adam algorithm, which control the exponential decay rates of the first and second moments, respectively; and $g_{clip}$ is the gradient at the current time.

Bias corrections are then made to the first-order moment estimation $\hat{m_{t}}$ and the second-order moment estimation $\hat{v_{t}}$ as

(35) $$\hat{m_{t}}=\frac{m_{t}}{1-\beta_{1}^{t}}$$

(36) $$\hat{v_{t}}=\frac{v_{t}}{1-\beta_{2}^{t}}$$

Finally, the model parameters updated as follows:

(37) $$g_{t}=g_{t-1}-\frac{\alpha }{\sqrt{\hat{v_{t}}}+\varepsilon }\hat{m_{t}}$$

Here, $g_{t}$ represents the model parameters at the current moment; $g_{t-1}$ represents the model parameters at the previous moment; α is the learning rate; and ε is a numerical stability constant.

### 2.5. Model Evaluation Indicators

To comprehensively quantify the algorithmic results and evaluate the performance of the pig-cough recognition model, this study adopted a multi-level evaluation framework, including basic classification metrics, statistical significance testing, model calibration analysis, and feature visualisation.

#### 2.5.1. Basic Classification Metrics

To measure the performance of the proposed model, four key indicators were employed: accuracy (*A*), precision (*P*), recall (*R*), and F1-score. Their expressions are as follows:

(38) $$A=\frac{TP+TN}{TP+TN+FP+FN}$$

(39) $$P=\frac{TP}{TP+FP}$$

(40) $$R=\frac{TP}{TP+FN}$$

(41) $$F1=\frac{2TP}{2TP+FP+FN}$$

Here, *TP* represents the number of cough samples correctly identified; *TN* denotes the number of non-cough samples correctly identified; *FP* represents the number of non-cough samples wrongly reported as cough; and *FN* denotes the number of cough samples wrongly reported as non-cough.

#### 2.5.2. Statistical Significance Testing

To validate the statistical reliability of model performance and ensure it surpasses random guessing levels, this study employed three evaluation approaches:

k-Fold Cross-Validation: The dataset is randomly partitioned into k (k = 5) mutually exclusive subsets of similar size. Each time, the union of k−1 subsets is used as the training set, and the remaining single subset is used as the validation set. This process is repeated for k rounds of training and validation, and the average of k evaluation results is considered as the final performance estimate. This method maximises data utilisation and provides a more stable and reliable assessment of the generalisation performance of the model.

Permutation Test: This test evaluates the statistical significance of model performance by disrupting the true relationship between labels and features. The specific steps are as follows: (a) The model performance score (e.g., accuracy) is recorded under true labels, denoted as S_true_; (b) the sample labels are randomly shuffled (permuted) multiple times (e.g., 100 times), and the model performance score is recalculated after each permutation, obtaining a distribution of scores S_perm,i_ under the null hypothesis that model performance is attributed to randomness; and (c) the *p*-value is calculated, which is the proportion of permuted scores that are greater than or equal to the true score: $p=\;\frac{count(S_{perm}\geq S_{true})+1}{N_{\mathrm{permutations}}+1}$, where $N_{\mathrm{permutations}}$ represents the total number of random permutation experiments conducted. If the *p*-value is less than the significance level (e.g., 0.05), the null hypothesis is rejected, indicating that model performance is statistically significant.

Confidence Intervals (CIs): Based on the results of k-fold cross-validation, the 95% CI for a performance metric (e.g., mean accuracy) is calculated using the t-distribution: $CI=\bar{x}\pm t_{\left(1-\alpha /2,df\right)}\times (s/\sqrt{k})$, where $\bar{x}$ is the mean, s is the standard deviation, α is the confidence interval, k is the number of folds, and df is the degrees of freedom (k−1). This interval provides a range for estimating the overall performance, and a narrower interval indicates a more precise estimate.

#### 2.5.3. Model Calibration and Uncertainty Analysis

Reliability Diagram and Expected Calibration Error (ECE): Model calibration measures whether the confidence level of its predicted probabilities matches the actual accuracy. This is evaluated by plotting a reliability diagram and calculating the ECE through binning: the predicted probability interval [0, 1] is divided into M equal-width intervals (bins). The calibration error for each bin is given as $ECE=\sum_{m=1}^{M}\frac{\left|B_{m}\right|}{N}|\mathrm{acc}(B_{m})-\mathrm{conf}(B_{m})|$, where $\mathrm{acc}(B_{m})$ is the sample accuracy within bin $B_{m}$, and $\mathrm{conf}(B_{m})$ is the average predicted probability within bin $B_{m}$. The ECE is the sum of calibration errors across all bins. A lower ECE value indicates better model calibration and more reliable predicted probabilities.

Area Under the Precision–Recall Curve (AUPRC): In datasets with class imbalance (such as cough event recognition in this study), the AUPRC is a more robust performance metric than the area under the receiver operating characteristic curve. It evaluates the ability of the model to identify the minority class (cough) by plotting the relationship between precision and recall at different decision thresholds and calculating the AUC. A higher AUPRC value indicates better model performance on imbalanced data.

## 3. Experimental Design and Result Analysis

### 3.1. Experimental Setup

To establish a fair and reproducible evaluation framework, we defined the foundational conditions of the experiments from four aspects—data annotation, dataset partitioning, comparison methods, and the experimental environment—providing a unified reference standard for subsequent result analysis.

#### 3.1.1. Data Annotation

To directly address the challenge of environmental noise interference and to construct a high-quality, discriminative dataset, this study employed a fine-grained audio event annotation protocol. This approach was chosen over signal-level filtering techniques, as the latter risk distorting the target cough’s acoustic features in a complex soundscape where noise and target signals exhibit significant spectral overlap. Two primary audio categories were defined: Target Cough Sounds and Environmental Noise. The noise category was further subdivided into: (1) Equipment Noise (e.g., continuous or intermittent sounds from feeders and ventilation systems), (2) Animal Activity Noise (e.g., sounds generated by pigs moving, rooting, or interacting with pen fixtures), and (3) Non-Cough Vocalizations (e.g., squeals or barks). All audio recordings were manually reviewed and annotated by experienced researchers, who identified coughs based on their distinctive acoustic patterns (e.g., short, explosive bursts with specific spectral contours) and concurrently labeled any segment containing the predefined environmental noises. Positive samples (“Cough”) were strictly extracted only from clear, uncontaminated segments without overlapping labeled noise. Conversely, the negative samples (“Noise”) were directly drawn from the annotated noise segments, thereby creating an adversarial dataset that represents real-world interference. This strategy compels the model to learn to distinguish the target signal directly from raw, unprocessed audio, significantly enhancing its robustness in complex acoustic environments.

#### 3.1.2. Dataset Partitioning

To objectively evaluate the generalisation ability of the model and ensure the statistical reliability of the results, this study adopted a strategy combining stratified cross-validation with a hold-out test set. Specifically, the entire dataset was first split into a training-validation set and an independent test at a ratio of 85:15. The test set was used solely in the final evaluation phase to ensure the objectivity of the assessment results. Building on this, a 5-fold stratified cross-validation was performed on the training set for model selection and hyperparameter tuning. More concretely, training data were randomly divided into five mutually exclusive subsets, each maintaining the original class distribution. In each validation round, four subsets (80%) were used as the training subset, and the remaining one subset (20%) served as the validation subset. This process was repeated iteratively until every subset had been used once as the validation set.

#### 3.1.3. Comparison Methods

To comprehensively validate the performance advantages of the proposed model, representative models covering both traditional machine learning and deep learning were selected as comparison baselines:

Traditional Machine Learning Baselines: SVMs and random forest (RF), known for their stable performance in small-sample scenarios, were selected. Features comprised 34-dimensional multi-features (including the MFCC and spectral centroid) extracted using Librosa (version 0.8.1).

Deep Learning Baselines: CNNs and long short-term memory (LSTM) networks were chosen to validate local feature extraction and sequence modelling capabilities of the model, respectively. These models also used the Librosa-based multi-features.

Proposed Model: The PANNs-CNN14-TFDS model, based on transfer learning, considered Log-Mel spectrograms as inputs and combined the local features from the pre-trained backbone network with the global relational features from the TFDS module.

#### 3.1.4. Experimental Environment

All experiments were conducted on a 64-bit Windows 11 computer configured with an 11th-Gen Intel Core i5-1135G7 processor (Intel Corporation, Santa Clara, CA, USA), Intel Iris Xe Graphics (128 MB dedicated memory; Intel Corporation, Santa Clara, CA, USA), and 16 GB RAM (Samsung Electronics Co., Ltd., Suwon, South Korea). The software environment was based on Python 3.8, utilising PyTorch 2.4.1 and PaddlePaddle 2.4.2 as deep learning frameworks. To ensure the reproducibility of results, we fixed all random seeds and enabled deterministic algorithms in both major frameworks, guaranteeing identical outputs for the same code and input across any number of runs.

### 3.2. Time-Domain and Frequency-Domain Characteristics

**Figure 6:**
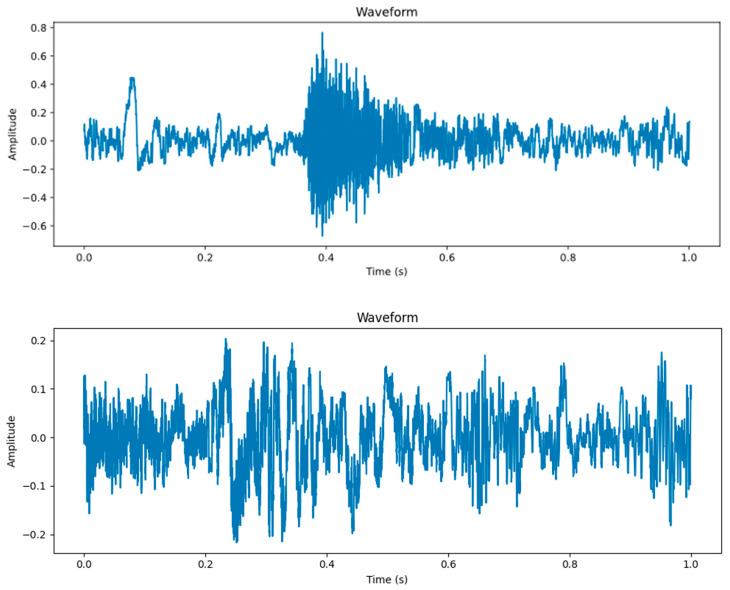
Time-frequency Features of Cough vs. Non-cough Signals.

The time-domain and feature maps of cough and non-cough signals are shown in Figure 6. In the time domain, cough signals exhibit impulse characteristics, typically showing sharp fluctuations in amplitude (±0.4) lasting 0.2–0.4 s. These signals form a steep rising edge and multiple decaying oscillation peaks, followed by a rapid return to a stable baseline state. This short burst of impulse pattern is attributed to the sudden release of air flow in the lungs and the physiological mechanism of glottis opening and closing, in sharp contrast to the continuous and smooth fluctuations of non-cough signals.

There are essential differences between cough and non-cough signals in terms of physiological acoustic mechanisms and temporal dynamic characteristics. The specific features are presented in Table 1.

**Figure 7:**
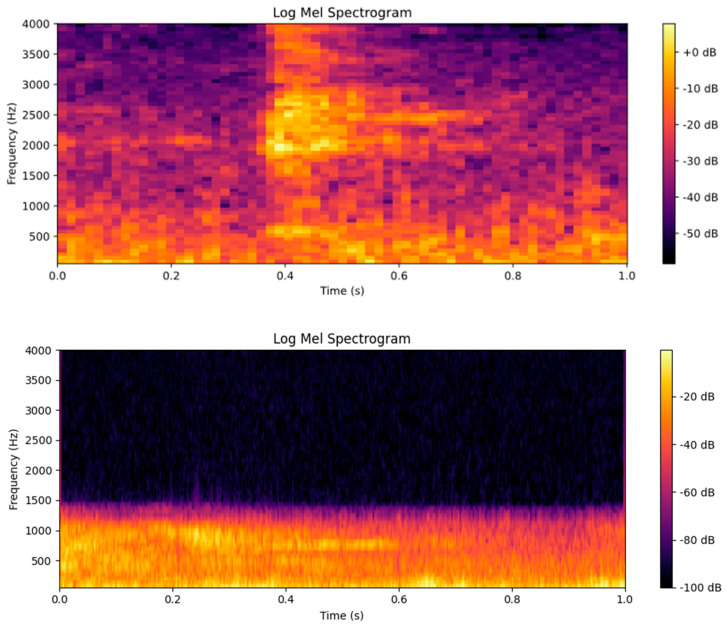
Log-Mel Spectrogram Feature Maps of Cough and Non-Cough Signals.

The differences in spectral energy distribution are visually verified in the Log-Mel feature maps (Figure 7). In the upper panel of Figure 7 (cough), energy is concentrated in the medium-to-high-frequency region, and high-frequency components persist. In the lower panel of Figure 7 (non-cough), low frequencies dominate, while high frequencies decay rapidly. This demonstrates that cough and non-cough signals exhibit significant differences in their log-Mel spectrogram features.

### 3.3. Multi-Model Performance Comparison and Generalisation Ability Verification

This section evaluates the performance of various models based on the independent test set, comparing them in terms of generalisation performance, training epochs, and training dynamics.

#### 3.3.1. Performance Comparison on the Independent Test Set

Two complementary approaches were used to compare model performance. On the one hand, the traditional Librosa-based multi-feature extraction algorithm (including 34-dimensional features such as the MFCC and spectral centroid) was used in combination with classic machine learning classifiers (SVM and RF) and deep learning models (CNN and LSTM). On the other hand, the Log-Mel spectrogram feature and the pre-trained PANNs-CNN14-TFDS model were applied. A systematic comparison was conducted through four types of quantitative indicators (accuracy, precision, recall, and F1-score) on a unified test set, and the results are presented in Table 2.

#### 3.3.2. Effect of Training Epochs

To examine the impact of training duration on model convergence, the performance of deep learning models was compared under two training regimes (50 and 100 epochs), and the results are presented in Table 3 and Table 4. For deep learning models, increasing the number of training epochs from 50 to 100 significantly improved the performance of both CNN and LSTM models. However, the recall rates of both models remained stable at 70.00%, indicating a bottleneck in the recognition ability of cough samples. By contrast, the combination of Log-Mel and PANNs-CNN14-TFDS consistently maintained the highest accuracy (94.74%) and a relatively large F1-score (>92%). This suggests that the transfer of pre-trained representations of the PANN in the large-scale audio domain can effectively overcome the limitations of small sample sizes.

#### 3.3.3. Analysis of Training Dynamics and Generalisation Ability

The training stability and generalisation ability of the models were analysed by examining loss and accuracy curves from the training and validation sets.

**Figure 8:**
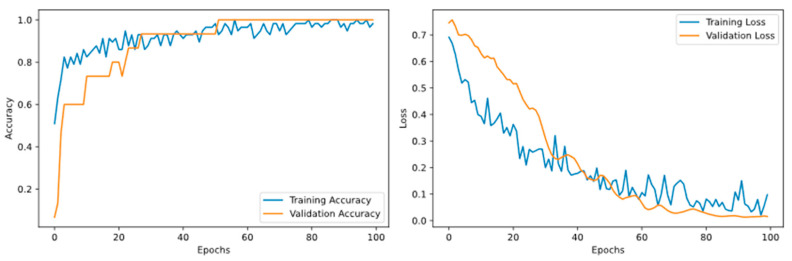
Accuracy and Loss Rates of the CNN Model on Training and Validation Sets.

CNN Model (Figure 8): Although the accuracy of the validation set approached 100%, the accuracy of the independent test set was only 85.71%. A noticeable divergence emerged between the training and validation curves in the later stages, indicating severe overfitting. This occurred because the CNN failed to learn the true underlying data distribution from the limited sample size, causing it to merely capture noise and local specifics present in the training data.

**Figure 9:**
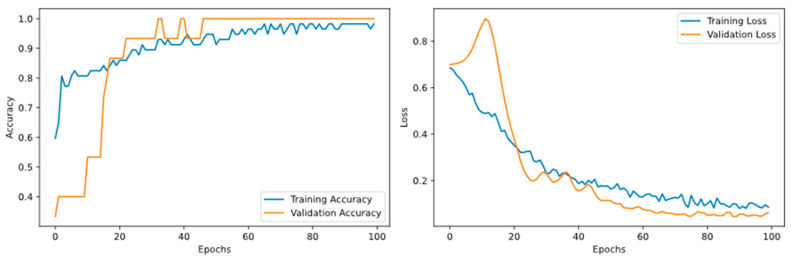
Accuracy and Loss Rates of the LSTM Model on Training and Validation Sets.

LSTM Model (Figure 9): Similar to the CNN model, the LSTM model exhibited insufficient generalisation capability during training. The performance gap between the training and validation sets progressively widened, ultimately resulting in poor performance on the test set. This result confirms that sequence-based architectures also face the challenge of overfitting in small-sample audio tasks.

**Figure 10:**
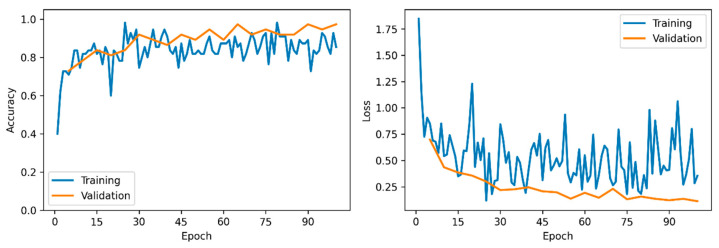
Accuracy and Loss Rates of the PANNs-CNN14-TFDS Model on Training and Validation Sets.

PANNs-CNN14-TFDS Model (Figure 10): The loss and accuracy curves for the training and validation sets remained closely aligned throughout the training process without significant divergence, and both stabilised in the later phases. This indicates that the proposed model did not experience overfitting. Combined with its high accuracy on the independent test set, these findings further confirm the robust generalisation ability of the model.

### 3.4. Reliability Verification

While the results from the independent test set reflect the final model performance, a single data split may introduce randomness. To assess the stability and statistical reliability of model performance, we employed 5-fold stratified cross-validation on the training set (i.e., the 85% dataset). The final reported mean values for metrics such as accuracy and F1-score, along with their 95% CIs, were calculated based on the results from these 5 validation folds. These results reflect the stability and generalisation capabilities of the model across different splits of the training data.

#### 3.4.1. Stability and Statistical Significance

**Figure 11:**
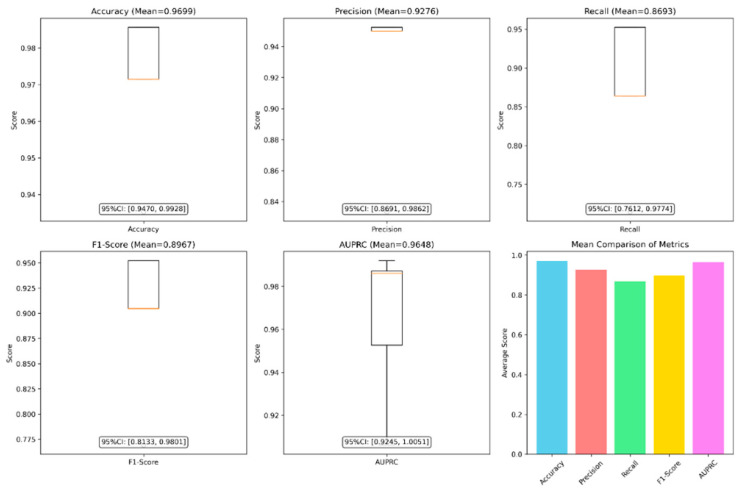
Cross-validation performance metrics with 95% confidence intervals. **Note:** Yellow line: median.

The metrics obtained from cross-validation demonstrated excellent and highly stable performance (see Figure 11). Specifically, the model achieved a mean accuracy of 96.99% (95% CI [0.9470, 0.9928]), indicating minimal performance fluctuation. Furthermore, the confidence intervals for precision (mean = 0.9276, 95% CI: [0.8691, 0.9862]), recall (mean = 0.8693, 95% CI: [0.7612, 0.9774]), and F1-score (mean = 0.8967, 95% CI: [0.8133, 0.9801]) were all positioned at high values and exhibited narrow ranges.

To validate the statistical significance of model performance, this study employed permutation tests to rigorously evaluate the classification effectiveness of the PANNs-CNN14-TFDS model. After 100 permutation experiments, the results demonstrated that the model achieved an accuracy of 0.9333 under true labels, while the average accuracy with randomly shuffled labels was only 0.7413, yielding a significant performance disparity of 19.2%. The statistical test yielded a *p*-value of less than 0.001, which exceeded the threshold for statistical significance.

These results robustly confirm that the PANNs-CNN14-TFDS model possesses exceptional stability and statistical reliability. Its performance remained consistent across different data subsets, demonstrating strong generalisation ability and effectively avoiding overfitting.

#### 3.4.2. Model Calibration Analysis

Model reliability depends not only on stable classification accuracy but also on the alignment between predicted probabilities and the actual class distribution (i.e., calibration), which is fundamental for trustworthy decision-making in real-world deployment. To comprehensively evaluate the combined performance of model calibration quality and classification effectiveness, this study performed an analysis integrating the reliability diagram and the precision–recall curve. This approach validated the accuracy of the predicted probabilities while further solidifying the superior classification performance of the model.

**Figure 12:**
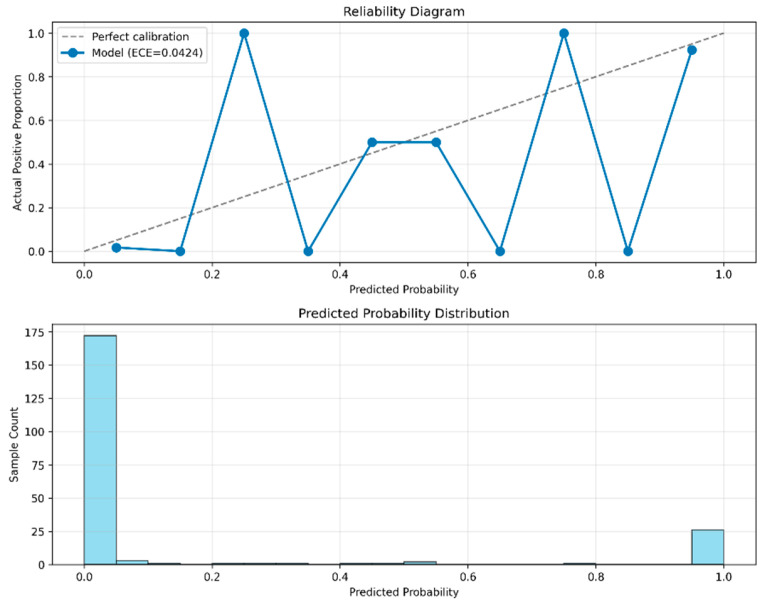
Calibration reliability diagram for the PANNs-CNN14-TFDS model.

As shown in Figure 12, the proposed PANNs-CNN14-TFDS model demonstrated a high level of calibration. The prediction curve closely followed the ideal calibration line, with an ECE of only 0.0424, indicating that the probability values outputted by the model are highly consistent with the actual positive class likelihood. Further observation of the predicted probability distribution revealed a distinct peak in the number of samples at both ends of the probability range, forming a characteristic U-shaped distribution. This result indicates that the model exhibits high confidence in its predictions for the majority of samples, assigning uncertain intermediate probabilities to only a few instances, thereby demonstrating clear decision boundaries and strong discriminative capability.

**Figure 13:**
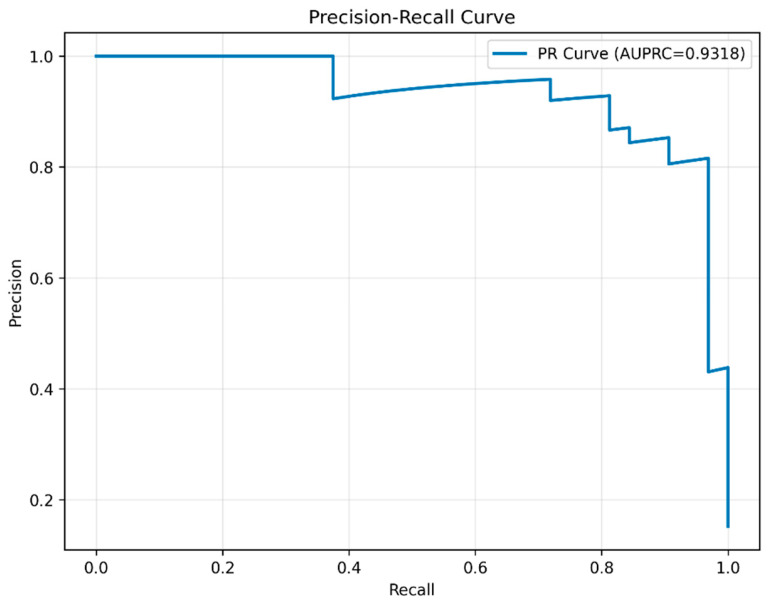
Precision–recall curve for the PANNs-CNN14-TFDS model.

In terms of classification performance, the precision–recall curve (Figure 13) further validated the comprehensive effectiveness of the model in handling positive-class samples. The curve demonstrated that a strong balance between precision and recall was maintained across different decision thresholds. The achieved AUPRC of 0.9318 indicated that, even within an imbalanced dataset context, the model can simultaneously sustain high precision and high recall, showcasing its excellent capability in identifying positive instances.

Furthermore, the PANNs-CNN14-TFDS model not only provided reliable probability estimates in a statistical sense but also demonstrated consistently superior classification performance at the task level. These findings confirm its high predictive reliability and practical value for real-world deployment.

### 3.5. Ablation Study

To clarify the necessity and contribution of each core component of the proposed model, four ablation variants were designed based on the baseline architecture. These variants enabled performance comparison through controlled single-variable modifications. All variants were trained and evaluated under strictly consistent experimental conditions, including identical train/test splits, optimizer (Adam), batch size, number of epochs, and early stopping strategy, to ensure a fair comparison and the reliability of conclusions.

#### 3.5.1. Design of Ablation Variants

The baseline model is structured as a frozen, pre-trained PANNs-CNN14 backbone network coupled with a TFDS module, a feature concatenation layer, and a classification head. A stratified learning rate (3 × 10^−4^ for the TFDS module; 1 × 10^−3^ for the classification head) is employed to account for the distinct learning dynamics of each component. This design embodies our complete proposal, aiming to validate the optimal performance attained through the synergistic integration of a frozen general-purpose acoustic backbone and a lightweight specialized time-frequency module via concatenation-based fusion.

The ablation variants were designed as follows:

Variant 1 (No TFDS): The TFDS module was removed, retaining only the PANNs-CNN14 backbone network, whose output was fed directly into the classification head. This variant aims to quantify the contribution of the TFDS module.

Variant 2 (No CNN14): The PANNs-CNN14 backbone network was removed, retaining only the TFDS module, whose output was fed directly into the classification head. This variant aims to quantify the contribution of the pre-trained backbone network.

Variant 3 (Fusion-Add): Both the PANNs-CNN14 and TFDS modules were retained, but the feature fusion method was changed from concatenation to element-wise addition. A linear layer was introduced to project the CNN14 output dimension to 128, aligning with the TFDS feature dimension before addition. This variant aims to compare the effectiveness of different feature fusion strategies.

Variant 4 (No Stratified LR): The model structure remained unchanged, but the stratified learning rate was abandoned, using a uniform learning rate of 3 × 10^−4^ for both the TFDS module and the classification head. This variant aims to validate the necessity of the stratified learning rate strategy.

#### 3.5.2. Results and Analysis of the Ablation Study

The performance comparison of ablation experiments is summarised in Table 5.

Comparison of Variant 1 with Baseline: Removing the TFDS module resulted in performance decreases of approximately 6% in accuracy, 31% in F1-score, and 29% in AUPRC. This underscores the critical role of the TFDS module in modelling time–frequency associative features, effectively capturing the synergistic patterns between temporal rhythm and spectral energy distribution in cough sounds.

Comparison of Variant 2 with Baseline: Removing the CNN14 backbone caused a decrease of approximately 2% in accuracy, 8% in F1-score, and 13% in AUPRC. This indicates that the local spectral features (e.g., energy peaks in specific frequency bands) provided by CNN14 serve as fundamental representations for the recognition task.

Comparison of Variant 3 with Baseline: Changing the feature fusion method from concatenation to element-wise addition caused significant decreases of approximately 14% in accuracy, 37% in F1-score, and 21% in AUPRC. This indicates that concatenation fusion more effectively preserves the independent information contained in both local details and global associations. By contrast, the additive operation likely caused information blending and diluted critical features, thereby validating the effectiveness of the concatenation strategy.

Comparison of Variant 4 with Baseline: Abolishing the stratified learning rate setting did not decrease the accuracy and F1-score, but the AUPRC decreased by approximately 5%. This suggests that the classification head still requires a higher learning rate to adapt to the distributional differences of the fused features. The stratified optimisation strategy aids model convergence and enhances the ability to identify positive instances.

The ablative studies have established design principles for the model across three key aspects: at the feature extraction level, the local spectral features provided by CNN14 and the global time-frequency associative features captured by the TFDS module complement each other, with both being indispensable; at the feature fusion level, the concatenation method outperforms element-wise addition by more effectively preserving information; and at the optimization strategy level, the stratified learning rate facilitates model convergence.

### 3.6. Hyperparameter Configuration Comparison

Having established the effectiveness of the model architecture, we now focus on optimizing the training process. Hyperparameter configuration plays a critical role in determining model performance and training efficiency. As summarized in Table 6, we evaluated various combinations of learning rates and regularization strategies.

The results indicate that employing a higher learning rate (0.001) substantially enhanced model performance while maintaining relatively short training times, especially when weight decay was omitted. When a lower learning rate was applied, the use of double regularization—combining weight decay with gradient clipping—led to improved performance and faster convergence. In contrast, under a high learning rate regime, regularization techniques—whether weight decay or gradient clipping—showed no significant effect on final performance; however, excluding weight decay in this scenario further reduced training time.

**Figure 14:**
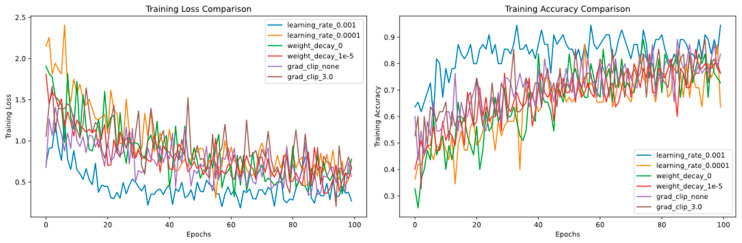
Changes in the Training Loss and Accuracy of the Model over Training Epochs under Different Hyperparameter Settings.

Further observations of the training dynamic features are illustrated in Figure 14 (left), demonstrating variations in training loss with epochs under different hyperparameter settings. The model with a learning rate of 0.001 (blue) exhibited the lowest training loss, indicating that a higher learning rate promotes rapid model convergence. By contrast, the model with a learning rate of 0.0001 (orange) had a higher loss value, suggesting that a lower learning rate slows down the convergence speed. Notably, although weight decay (λ = 10^−5^, red) and gradient clipping (τ = 0.3, brown) are theoretically regularisation methods, their impact on loss reduction was minimal in this experiment. In particular, the weight decay group yielded slightly higher loss than the non-regularised group.

This trend was verified in the accuracy dimension. In Figure 14 (right), changes in training accuracy correspond to loss changes. The model with a high learning rate consistently maintained superior accuracy, whereas that with a low learning rate improved more slowly. Moreover, the accuracy curves of the regularised and non-regularised groups were nearly identical, further confirming that weight decay and gradient clipping did not produce the expected effect in this task.

**Figure 15:**
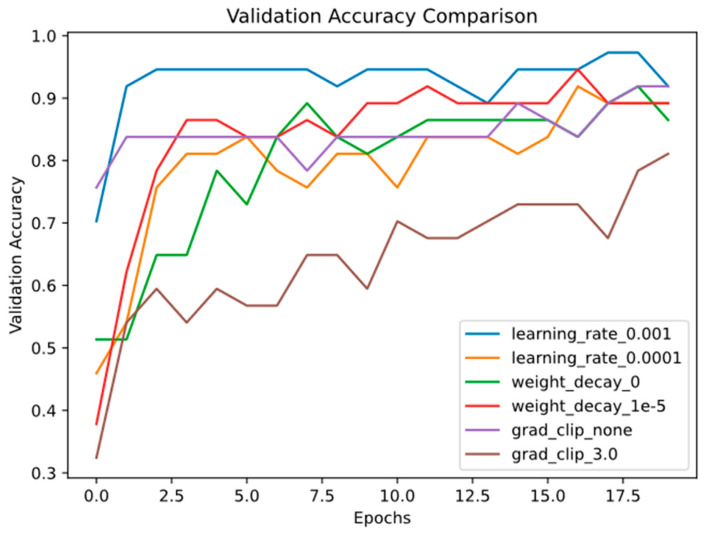
Changes in the Validation Accuracy of the Model over Training Epochs under Different Hyperparameter Settings.

In the experiment, we found that conducting validation every five epochs proved effective for evaluating model performance. This approach helped detect early signs of overfitting in the model and enabled dynamic adjustment of training strategies, thereby achieving better generalisation performance on the final test set. Figure 15 shows the accuracy of the validation set under different parameter settings. We found that a higher learning rate accelerated learning and led to peak performance more rapidly. However, weight decay and gradient clipping had limited influence on model performance.

## 4. Discussion

Accurate and timely recognition of pig coughs is crucial for the early detection of respiratory diseases in intensive farming systems, as it enables targeted interventions to reduce mortality and antibiotic use [2]. Previous studies have explored various approaches for pig cough recognition, ranging from traditional machine learning methods to deep learning models. However, challenges persist in adapting these methods to complex farm environments [23] and small-sample scenarios [24]. Kong et al. demonstrated the effectiveness of PANNs in audio pattern recognition tasks, particularly when the training data are limited. Through pre-training and transfer learning, PANNs can achieve or approach state-of-the-art performance across a variety of tasks [12]. In this context, the present study proposed a transfer learning strategy based on the PANNs-CNN14-TDFS model to overcome the ’small-sample generalisation bottleneck’. The model achieved superior performance compared with conventional models, providing new insights into acoustic-based disease monitoring in livestock.

### 4.1. Advantages of the PANNs-CNN14-TDFS Model in Pig Cough Recognition

Traditional machine learning models such as SVM and RF have been widely used in pig cough recognition; however, their performance is constrained by manual feature engineering and sensitivity to noise [9,10]. For example, SVM models relying on Librosa-extracted features (e.g., MFCCs) often struggle with overlapping time–frequency components between coughs and farm noise, leading to low recall rates for true cough events (Table 3). These findings are consistent with those of Ferrari et al. [3], who noted that environmental noise in pig farms significantly degrades the performance of traditional acoustic classifiers.

Deep learning models such as CNN and LSTM have demonstrated improved feature extraction capabilities; however, they experience overfitting in small-sample scenarios [25]. In the present study, the CNN model achieved near-perfect accuracy on the validation set but dropped to 85.71% on the test set (Table 4), indicating it had overfit to training noise. Similarly, the LSTM model, despite its ability to capture temporal dependencies, failed to generalise effectively due to insufficient training data, yielding an F1-score of only 73.68% (Table 4). This is consistent with the observations of Schmidt et al. [26], who reported that deep neural networks without pre-training often struggle to learn robust features from limited samples.

In contrast, the PANNs-CNN14-TFDS model, capitalizing on transfer learning, attained an accuracy of 94.74% and an F1-score of 92.31% (Table 4), surpassing all compared models. This performance advantage is attributed not only to its pre-trained backbone—inheriting general acoustic representations from AudioSet—but also to the TFDS module’s capacity to capture critical time-frequency dependencies. Together, they allow the model to identify intricate acoustic patterns (e.g., the 0.2–0.4-s impulse structure of pig coughs in Figure 6) with limited fine-tuning samples, consistent with findings in [13]. The lightweight fine-tuning strategy, updating only the FC layer, further reduced overfitting and ensured robust generalization amid unseen farm noise.

### 4.2. Impact of Model Parameters on Performance

Hyperparameter tuning is critical for optimising model performance, especially in small-sample scenarios. Our results showed that a higher learning rate (0.001) significantly accelerated model convergence, with the training loss reaching a lower plateau faster than with a lower learning rate (0.0001) (Figure 14). This aligns with the findings of Ma et al. [18], who reported that appropriate learning rates can enhance the adaptability of deep models to a new task.

Notably, weight decay and gradient clipping—commonly used to prevent overfitting—had limited impact on model performance in our experiment (Table 6). This may be attributed to the transfer learning paradigm: the pre-trained and frozen CNN14 backbone provides robust and generalisable acoustic representations, while the TFDS module explicitly models global time-frequency interactions—a form of structural inductive bias that reduces the reliance on explicit regularisation.

### 4.3. Role of Log-Mel Spectrograms in Cough Feature Extraction

The choice of acoustic features directly influences model performance. In this study, we adopted Log-Mel spectrograms to capture the time–frequency characteristics of pig coughs, which exhibit distinct high-energy bursts in the 1000–3000 Hz range (Figure 7). This feature engineering step effectively highlights the impulse-like structure of coughs, distinguishing them from low-frequency environmental noise (e.g., ventilation systems).

Compared with traditional features such as MFCC, Log-Mel spectrograms preserve more high-frequency details, which are essential for identifying short-duration cough pulses (0.2–0.5 s) (Figure 6). This is consistent with that reported by Zhu et al. [27], who demonstrated that time–frequency features retaining high-frequency components improve the recognition of transient acoustic events in livestock. This feature choice synergizes with the PANNs-CNN14-TFDS network to form a comprehensive analysis framework: Log-Mel spectrograms supply the rich time-frequency map, the CNN14 backbone decomposes it into local spectral features, and the TFDS module elevates this by capturing long-range dependencies and synergistic patterns between temporal and frequency dimensions. Together, they enable a hierarchical understanding of cough sounds, from local details to global associative structures.

### 4.4. Limitations and Future Directions

Despite the promising results, this study has several limitations. First, the dataset was collected from a single farm (JiaHeng Pig Farm) and included a small sample size (107 cough events and 590 noise segments). In particular, the cough samples originated from only one pig, which may introduce individual bias and constrain the model’s ability to generalize to other animals with different acoustic characteristics. This may further restrict the generalisability of the model to other farms with different noise profiles (e.g., varying ventilation or feeding equipment). Therefore, future work should prioritize expanding the dataset to include cough samples from more individuals across diverse breeds, ages, and health statuses. This will be essential for rigorously evaluating and improving the model’s robustness and applicability in real heterogeneous pig populations.

Second, the model focuses solely on acoustic features, without integrating visual or environmental data (e.g., temperature and humidity). Integrating multi-modal data could further improve recognition accuracy, as suggested by studies showing that combining audio and video enhances behavioural anomaly detection in pigs [28]. In subsequent research, we plan to construct an audio-visual fusion model to achieve cross-validation and precise localization of sound source.

Third, the current model does not distinguish between different types of coughs (e.g., those caused by bacterial vs. viral infections). Future research could explore fine-grained classification by incorporating pathological labels, enabling more precise disease diagnosis.

Furthermore, future studies should systematically compare the proposed framework with state-of-the-art audio classification models, including Transformer-based architectures, on larger-scale and multi-scenario datasets. Such comparisons would help further delineate the performance boundaries and practical potential of the method.

### 4.5. Practical Implications

This study demonstrated the feasibility and potential of pre-trained audio models in agricultural applications. By reusing general acoustic knowledge from large-scale datasets, the PANNs-CNN14-TFDS model overcomes the challenge of the limited number of annotated samples in livestock farms, providing a cost-effective solution for real-time cough monitoring. For farmers, this technology enables the early detection of respiratory diseases, reducing economic losses and promoting welfare-orientated farming practices.

Moreover, the transfer learning strategy proposed here offers a methodological reference for other small-sample audio recognition tasks in agriculture, such as recognising chicken distress calls or cattle vocalisations. It highlights the value of leveraging pre-trained models to bridge the gap between large-scale general datasets and domain-specific small-sample problems.

In conclusion, the PANNs-CNN14-TFDS model with transfer learning represents a significant advancement in pig cough recognition, particularly under small-sample conditions. Its superior performance stems from synergy between pre-trained acoustic features, Log-Mel spectrogram engineering, and lightweight fine-tuning. Future work focusing on multi-farm validation, multi-modal integration, and fine-grained classification will further enhance its practical utility in livestock health monitoring.

## 5. Conclusions

This study aimed to design a classification model algorithm for small-sample data. To optimise model performance, we conducted detailed hyperparameter tuning on the PANNs-CNN14-TDFS model, including the learning rate, weight decay, and gradient clipping. The experimental results indicated that a higher learning rate (0.001) enabled the model to converge to a lower loss value more rapidly during training, whereas a lower learning rate (0.0001) slowed down the convergence speed. Additionally, weight decay and gradient clipping had a limited impact on improving model performance in this experiment. These findings provide valuable insights for future model optimisation.

By comparing SVM, RF, CNN, LSTM, and PANNs-CNN14-TDFS, we found that the method combining Log-Mel spectral features with the PANNs-CNN14-TFDS model achieved the best performance on the test set, achieving an accuracy of 94.59%, precision of 87.67%, recall of 92.31%, and F1-score of 92.86%. This finding indicates that the deep learning method based on pre-training and fine-tuning has significant advantages in processing small-sample audio signals. The proposed model improved the recognition accuracy of 0.2–0.5 s cough pulses by 5.12% compared with the SVM model and by 10.81% compared with the CNN model.

This study experimentally verified the effectiveness and feasibility of deep learning models based on pre-training and fine-tuning in pig cough recognition. The proposed framework can assist farmers in the early detection and management of respiratory diseases in pigs, thereby supporting technology-based, welfare-orientated pig farming. Moreover, this study also demonstrated the potential of pre-trained audio models in agricultural applications, providing a methodological and technical foundation for future research and having significant scientific value and application prospects.

## Figures and Tables

**Table 1:** Comparison of Characteristics between Cough and Non-Cough Signals.

Feature Dimension	Cough Signal Feature	Non-Cough Signal Feature
Physiological acoustic mechanism	Sudden release of air flow in the lungs (broadband noise), glottal opening and closing (pulse sequence), and airway turbulence (high-frequency noise).	The lack of a physiological vocalisation mechanism is manifested as the steady-state characteristics of environmental noise.
Dynamic characteristics of time	Sudden pulse structure (duration 0.1–0.3 s), clear time boundaries, and multiple energy peaks.	Continuous and stable energy distribution and the lack of clear start and end boundaries, with no significant pulse characteristics.
Spectral energy distribution	Manifests as a vertical bright band, indicating a short-lived broadband energy burst with most of the energy concentrated in the 1000–3000 Hz band.	Typically appears as a horizontal, band-like energy distribution, indicating that the energy is relatively continuous over time and concentrated in the low-to-mid frequency range (100–2000 Hz).

**Table 2:** Performance Comparison of the Model. %.

Feature Extraction Algorithm	Model	A	P	R	F1-Score
Librosa	SVM	89.47	100	71.43	83.33
Librosa	RF	91.89	81.25	100	89.66
Librosa	CNN	83.78	81.82	69.23	75.00
Librosa	LSTM	81.08	71.43	76.92	74.07
Log-Mel	PANNs-CNN14-TFDS	94.59	87.67	92.31	92.86

**Table 3:** Evaluation Based on 50 Training Rounds. %.

Feature Extraction Algorithm	Model	A	P	R	F1-Score
Librosa	CNN	82.14	77.78	70.00	73.68
Librosa	LSTM	75.00	63.64	70.00	66.67
Log-Mel	PANNs-CNN14-TFDS	94.74	87.50	100	93.33

**Table 4:** Evaluation Based on 100 Training Rounds. %.

Feature Extraction Algorithm	Model	A	P	R	F1-Score
Librosa	CNN	85.71	87.50	70.00	77.78
Librosa	LSTM	82.14	77.78	70.00	73.68
Log-Mel	PANNs-CNN14-TFDS	94.74	100	85.71	92.31

**Table 5:** Performance Comparison of Ablation Experiments.

Model Variant	Accuracy	F1-Score	AUPRC
Baseline	0.9523	0.8571	0.9074
Variant 1 (No TFDS)	0.8952	0.5926	0.6441
Variant 2 (No CNN14)	0.9333	0.7878	0.7886
Variant 3 (Fusion-Add)	0.8190	0.5365	0.7154
Variant 4 (No Stratified LR)	0.9523	0.8571	0.8620

**Table 6:** Performance Comparison under Different Hyperparameter Settings.

Experimental Group	Learning Rate	Weight Decay	Gradient Clipping	Feature Description	Best Validation Accuracy Rate (%)	Training Time (s)
1	0.0001	λ = 10^−5^	τ = 0.3	Reference	89.19	17.2
2	0.0001	λ = 10^−5^	None	No clipping	86.49	24.1
3	0.0001	λ = 0	τ = 0.3	No decay	83.78	23.8
4	0.0001	λ = 0	None	No regul.	81.08	24.0
5	0.001	λ = 10^−5^	τ = 0.3	High LR	94.59	23.8
6	0.001	λ = 10^−5^	None	High LR, no clip	94.59	23.7
7	0.001	λ = 0	τ = 0.3	High LR, no decay	94.59	14.3
8	**0.001**	**λ = 0**	**None**	**High** **LR,** **no** **regul** **.**	**94.59**	**14.2**

Note: LR: learning rate; Regul.: regularization; Best values are in bold.

## Data Availability

The original contributions presented in this study are included in the article. Further inquiries can be directed to the corresponding author.
